# Risk Factors for Chronic Kidney Disease in Adult Patients with Congenital Heart Disease and Its Relationship with Cardiovascular Mortality

**DOI:** 10.3390/jcm13226963

**Published:** 2024-11-19

**Authors:** Efrén Martínez-Quintana, Fayna Rodríguez-González

**Affiliations:** 1 Cardiology Service, Complejo Hospitalario Universitario Insular-Materno Infantil, Avd. Marítima del Sur s/n, 35016 Las Palmas de Gran Canaria, Spain; 2 Department of Medical and Surgical Sciences, Faculty of Health Sciences, Universidad de Las Palmas de Gran Canaria, 35016 Las Palmas de Gran Canaria, Spain; 3 Hospital Universitario de Gran Canaria Dr. Negrín, 35010 Las Palmas de Gran Canaria, Spain

**Keywords:** congenital heart disease, chronic kidney disease, renal failure, cyanosis, survival, adult, cardiovascular mortality

## Abstract

**Background**: Patients with congenital heart disease (CHD) show risk factors for chronic kidney disease (CKD) and it is well known that CKD has a large negative impact on survival. **Methods**: Observational and prospective cohort study. Adult CHD patients and controls were matched for age and sex. **Results**: A total of 657 CHD adult patients (cases) and 1954 controls were studied. Median age in CHD patients was 30 (17–62) years and 373 (57%) were male. The prevalence of CKD (Glomerular filtration rate (GFR) < 60 mL/min/1.73 m^2^) was 0.2% and 4.5% in the control and CHD groups, respectively. Binary logistic regression analysis determined as risk factors for CKD in CHD patients: age [1.54 (1.04–1.28), *p* = 0.009], dyslipidemia [19.8 (1.35–301.1), *p* = 0.031], low iron concentration [0.96 (0.96–0.93), *p* = 0.048], cyanosis [25.7 (1.60–411.8), *p* = 0.022], and Down syndrome [46.8 (8.09–2710), *p* = 0.003]. During a follow-up time of 6.8 (1.2–10.5) years, cardiovascular mortality occurred in 31 patients with CHD showing, through the Kaplan–Meier test, a worse outcome among patients with CKD (*p* < 0.05) as was also seen in the univariate Cox regression survival analysis. However, after adjusting for other variables, this significance was lost, with age remaining as the unique independent prognostic factor. **Conclusions**: The prevalence of CKD was much higher in patients with CHD than in the control group; age, cyanosis, and Down syndrome were the predictors of a higher risk of CKD among CHD patients. Although CKD was associated with worse survival in CHD patients, only age was identified as an independent prognostic factor for cardiovascular mortality.

## 1. Introduction

Approximately 1 in 100 (1%) live births have a congenital heart defect, making it one of the most common congenital anomalies [1]. In fact, the prevalence of adult patients with congenital heart disease (CHD) has increased due to advances in medical and surgical management with about 85–90% of children born with CHD now surviving into adulthood. Moreover, due to the improved survival rates, the number of adults living with CHD in high income countries has surpassed the number of children with these conditions.

Chronic kidney disease (CKD) in patients with CHD is a progressive condition with multifactorial causes such as increasing age [2], heart failure, arrhythmias, hemodynamic changes, cardiovascular risk factors, cardiac surgeries [3], infections [4], nephrotoxic drugs, or chronic hypoxemia [5,6]. It is a bi-directional relationship where dysfunction in either the heart or kidneys worsens the function of the other. Researchers have previously proposed a decline in cardiac output with decreased renal perfusion as the leading underlying cause for worsening kidney function. However, other potential mechanisms include neurohormonal system activation, which leads to a reduced forward flow and renal perfusion. Also, increased central venous pressures seem to be a critical factor [7]. In fact, when patients develop fluid overload due to heart failure, venous pressure increases and is transmitted back to the efferent arterioles, which result in a net decrease in the glomerular filtration pressure and renal injury [8].

Also, CKD causes a systemic and chronic proinflammatory state resulting in atherosclerotic lesions, calcification, and fibrosis, favoring an accelerated aging of the cardiovascular system. In fact, patients with CKD have high cardiovascular risk, with cardiovascular death being the leading cause of death [9]. As renal function is intricately linked to cardiovascular health, and impairment in renal function significantly increases the risk of cardiovascular death in the general population; we hypothesize that among patients with CHD, renal impairment also has a negative impact on cardiovascular outcome.

The objective of this study is firstly to assess the differences in renal function between patients with CHD and a control population; secondly, to determine the factors associated with a higher risk of CKD in patients with CHD; and finally, to know if CKD is associated with a higher risk of cardiovascular mortality among patients with CHD.

## 2. Method

This study followed a prospective, observational, and analytic cohort design. The case group comprised adult patients with CHD, aged 18 years or older, who were consecutively recruited from a single CHD outpatient clinic between January 2007 and December 2018. The control group consisted of individuals over 18 years of age who visited community health centers in the same geographical area between July 2017 and December 2018 for preventive health services. Controls were matched to CHD patients based on age and gender. Patients who did not provide written informed consent or had undergone surgery or hospitalization within the last six months were excluded from the study. Ethical approval was granted by the hospital’s research ethics committee.

### 2.1. Clinical Data

CHD diagnosis was verified using imaging modalities, primarily echocardiography, and the anatomical severity was categorized as simple, moderate, or complex [10]. Cardiovascular risk factors—including hypertension, diabetes, dyslipidemia, and smoking habits—were identified according to established criteria [11]. Body mass index (BMI) was calculated by dividing the patient’s weight in kilograms by the square of their height in meters (BMI = kg/m^2^). The glomerular filtration rate (GFR) was calculated using the Modification of Diet in Renal Disease (MDRD) formula [12]. GFR categories were assigned as stages 1 through 5 and classified according to the following values: G1: GFR higher than 90 mL/min/1.73 m^2^; G2: GFR between 60 to 89 mL/min/1.73 m^2^; G3a: GFR between 45 to 59 mL/min/1.73 m^2^; G3b: GFR between 30 to 44 mL/min/1.73 m^2^; G4: GFR between 15 to 29 mL/min/1.73 m^2^; and G5: GFR less than 15 mL/min/1.73 m^2^ or treatment by dialysis. Chronic kidney disease (CKD) was characterized by the presence of an estimated glomerular filtration rate (eGFR) of less than 60 mL/min/1.73 m^2^, persisting for 3 months or more, irrespective of the cause [13]. Medical treatments, such as anticoagulants, antiplatelets, antihypertensive agents, and iron supplements, were recorded. Mechanical valve prostheses carriers were gathered through medical record review. Systemic ventricular dysfunction was assessed via echocardiography, with left ventricular ejection fraction < 40% or a tricuspid annular plane systolic excursion (TAPSE) < 17 mm if the right ventricle supported systemic circulation [14]. Patients were classified as cyanotic if hemoglobin oxygen saturation was under 90%. Pulmonary hypertension was defined by a mean pulmonary arterial pressure ≥ 20 mmHg at rest [15] in a right heart catheterization. Patients with CHD were cataloged with Eisenmenger syndrome if they had pulmonary arterial hypertension with reversal of a left-to-right shunt to a right-to-left one with secondary cyanosis.

### 2.2. Blood Tests

Blood samples were collected for research after participants fasted for at least 10 h, and written informed consent was obtained. Analytes were measured using spectrophotometry with the Olympus AU 2700 system (Olympus Diagnostic, Hamburg, Germany). Hs-CRP levels were determined using the immunoturbidimetric method on the same biochemistry analyzer, with reagents and calibrators applied as per the manufacturer’s specifications. NT-pro-BNP levels were quantified using an immunoassay with the Siemens Stratus CS Acute Care Diagnostic System (Siemens Healthcare Diagnostics, Inc., Newark, DE, USA). The same equipment and reference ranges were used for both CHD patients and controls.

### 2.3. Follow-Up

Cardiovascular mortality was defined as death resulting from any cardiovascular cause such as myocardial infarction, stroke, heart failure, or sudden cardiac death. Deaths were categorized based on clinical records, autopsy reports (when available), and the primary cause listed on the death certificate, following the International Classification of Diseases (ICD) codes related to cardiovascular disease. Follow-up duration was recorded from the study’s onset until the occurrence of cardiovascular death.

### 2.4. Statistical Analysis

In the statistical analysis, both the mean and median were used to describe the central tendency of continuous variables. The mean was applied to normally distributed data, providing the average value, while the median was used for non-normally distributed data, reflecting the midpoint of the dataset with the 5th to 95th percentile range. To compare continuous variables between groups, two statistical tests were employed: Student’s *t*-test and the Wilcoxon rank-sum test. The *t*-test was used for normally distributed data to compare the means between two groups. For non-normally distributed data, the Wilcoxon rank-sum test (a non-parametric alternative) was applied to compare the medians, as it does not assume a normal distribution. Pearson’s chi-square test was the statistical test for categorical data. Binary logistic regression method was used to assess the relationship between a binary outcome (presence or absence of CKD) and one or more independent variables (e.g., cardiovascular risk factors, age, BMI, or blood test variables). The logistic regression model estimated the odds ratio (OR) for each independent variable, representing the odds of the outcome occurring in relation to the exposure. The results were presented as adjusted ORs with 95% confidence intervals (CIs) to indicate the strength and direction of associations. Multivariable logistic regression was performed to adjust for potential confounding variables, such as age, gender, and other relevant risk factors. The Kaplan–Meier log rank test was used to analyze the time until cardiovascular death occurred. Meanwhile, Cox regression survival analysis was used for investigating the effect of several variables upon the time cardiovascular death took place. In the univariate analysis, one parameter was varied at a time, whilst in the multivariate analysis, the variables that were significant in the univariate analysis were varied simultaneously. Cox regression generated hazard ratios (HRs) which were interpreted with a 95% CI. In. A *p*-value of <0.05 was considered statistically significant. Statistical Package for the Social Sciences (SPSS 24, Chicago, IL, USA) was used for data analysis.

## 3. Results

A total of 657 patients diagnosed with CHD provided informed consent and were included in the study after being monitored at our outpatient clinic. The control group comprised 1954 individuals. Based on the complexity of their anatomical conditions, patients were categorized into three groups: simple defects [351 patients (54%)], moderate defects [189 patients (29%)], and severe defects [117 patients (18%)]. Table 1 details the different types of CHD included in our study according to their anatomical complexity. Among CHD patients, 28 (4.3%) were carriers of mechanical valve prostheses, 108 (16.4%) had systemic ventricular dysfunction, 53 (8.1%) presented cyanosis, 43 (6.5%) had pulmonary arterial hypertension, 37 (5.6%) had Eisenmenger syndrome, and 42 (6.4%) had Down syndrome. Among Down syndrome, 8 out of 37 (21.6%) patients had Eisenmenger syndrome.

### 3.1. Clinical and Blood Test Data of Patients with CHD and the Control Population

From a clinical perspective, the prevalence of smokers was notably higher in the control group compared to patients with CHD. On the contrary, CHD patients were more likely to be hypertensive, diabetic, or dyslipemic. When it came to medication use, CHD patients were more likely to be prescribed antiplatelet agents, oral anticoagulants, beta-blockers, angiotensin-converting enzyme (ACE) inhibitors, angiotensin receptor blockers (ARBs), calcium channel blockers, oral iron, and loop diuretics than those in the control group (*p* < 0.05). Conversely, no significant differences were observed in the usage of statins between the two populations. As indicated in Table 2, patients with CHD exhibited significantly higher creatinine, hemoglobin, and bilirubin concentration compared to the control group. In contrast, the control group had significantly higher cholesterol levels. No significant difference was found in hs-CRP levels between both groups. The prevalence of CKD in the control group was 0.2% (3 patients) while in patients with CHD reached 4.6% (30 patients) (*p* < 0.001). Among patients with CHD, 391 (59.5%) were in stage 1 (>90 mL/min), 236 (35.9%) in stage 2 (60–89 mL/min), 23 (3.5%) in stage 3a (45–59 mL/min), 6 (0.9%) in stage 3b (30–44 mL/min), and 1 (0.2%) patient in stage 4 (15–29 mL/min) according to the CKD categories.

### 3.2. Clinical and Blood Test Data of CHD Patients Based on CKD

CHD patients with CKD were significantly older, males, had a higher BMI, and exhibited a greater prevalence of complex CHD defects, arterial hypertension, diabetes mellitus, and dyslipidemia compared to those without it (*p* < 0.05). Additionally, individuals with lower GFR levels experienced a poorer New York Heart Association (NYHA) functional class than their counterparts.

In terms of laboratory findings, CHD patients with CKD presented significantly lower iron levels when compared to those without it. In contrast, NT-pro-BNP and hs-CRP levels were significantly higher among congenital patients with worse renal function. Furthermore, as displayed in Table 3, patients with lower renal function showed an increased use of oral anticoagulants, beta-blockers, ARBs, loop diuretics, statins, and oral iron supplements while presenting an increased prevalence of cyanosis, pulmonary arterial hypertension, and Down syndrome than CHD patients with a GFR above 60 mL/min.

### 3.3. Predictors of a CKD in Patients with CHD

Table 4 outlines the factors associated with CKD in patients with CHD. The findings from the logistic regression analysis are displayed as both unadjusted (or crude) odds ratios, derived from individual variable models, and adjusted odds ratios reflecting a comprehensive model that includes all significant variables. According to the adjusted odds ratio column in the table, the following factors were identified as predictors of CKD in the CHD cohort: age [1.54 (1.04–1.28), *p* = 0.009], dyslipidemia [19.8 (1.35–301.1), *p* = 0.031], iron concentration [0.96 (0.96–0.93), *p* = 0.048], cyanosis [25.7 (1.60–411.8), *p* = 0.022], and Down syndrome [46.8 (8.09–2710), *p* = 0.003].

### 3.4. Cardiovascular Mortality in Patients with CHD

**Figure 1:**
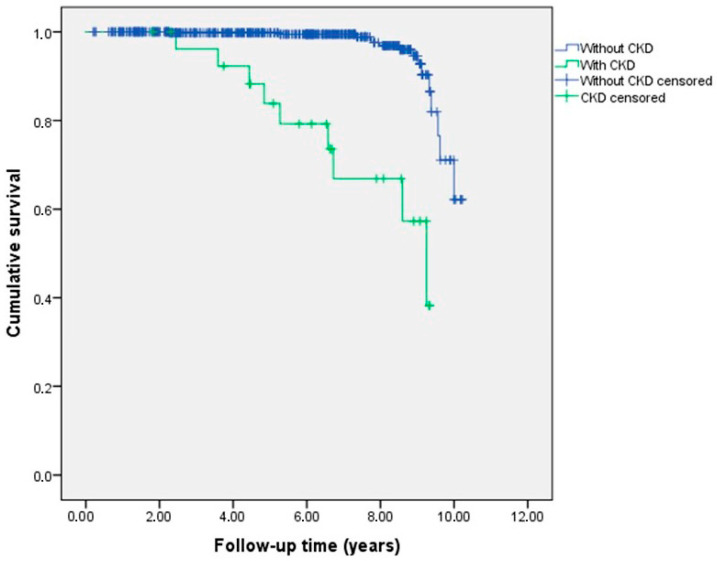
Kaplan–Meier curve shows cardiovascular mortality in congenital heart disease (CHD) patients with and without chronic kidney disease (CKD) (*p* < 0.001 for log-rank test).

Patients with CHD were monitored over a median follow-up period of 6.81 years (ranging from 1.17 to 10.46 years). During this time, 31 (4.7%) patients experienced cardiovascular mortality. Significant differences were observed in the rates of cardiovascular mortality (*p* < 0.001) between patients with and without CKD, as shown in Table 3. Additionally, the Kaplan–Meier survival curve depicted in Figure 1 illustrates that CHD patients with CKD had considerably poorer outcomes compared to those with higher GFR levels, with a log-rank test result of *p* < 0.001.

The univariate and multivariate Cox proportional hazards regression models were performed to identify independent risk factors for cardiovascular mortality among patients with CHD (Table 5). Univariate analysis showed that the risk factors of cardiovascular mortality were being older, having a complex CHD, having a NYHA functional class ≥ 2, presenting NT-pro-BNP levels above 125 pg/mL, and having CKD. However, multivariate analysis showed that the only predictor of cardiovascular mortality was age [HR = 1.05 (95% CI 1.02–1.08), *p* = 0.002].

## 4. Discussion

### 4.1. Prevalence

The prevalence of renal failure in patients with CHD can vary significantly based on several factors, including the type and severity of heart defects, comorbidities, cyanosis, hemodynamic compromise, or side effects secondary to surgical or percutaneous interventions [16,17]. Gillesén et al. [18] found that the risk of developing chronic kidney disease was 6.4 times higher in patients with CHD compared to controls, although with a lower prevalence (0.5%) than that reported by Dimopoulos et al. [19] where 9% of their patients with CHD had a GFR of <60 mL/min per 1.73 m^2^. In our series, intermediate results were obtained between the two previous studies yielding a CKD prevalence of 4.6% among patients with CHD, which is much higher than the 0.2% found in our age- and sex-matched control population. Nonetheless, we must take into account that Gillesen’s study included patients between 0 and 47 years old, while in our series and in that of Dimopoulos et al., only adult patients were included.

### 4.2. Risk Factors for CKD in Patients with CHD

In relation to renal function in patients with CHD, being older, male, and having associated cardiovascular risk factors was associated with a worse renal function, as also occurs in the general population. Age was associated with a worse renal function because as you get older, so do your kidneys. Men seem to be at an increased risk of renal failure sooner than women because of differences in hormone levels [20]. Obesity is a factor as overweight patients frequently exhibit elevated cardiovascular risk factors. Arterial hypertension is a factor as high blood pressure may constrict, narrow, and reduce the blood flow to the renal arteries leading to renovascular disease [21]. Diabetes mellitus is a factor as chronic hyperglycemia may result in the progressive damage of the renal microvasculature, which implies compromised filtration and renal dysfunction [22]. Dyslipidemia is a factor as it is strongly related to oxidative stress, inflammation, metabolic reprogramming, and fibrosis in renal tissues [23]. However, cardiovascular risk factors, age, or gender do not seem to be the only factors related to renal dysfunction in patients with CHD [24] as medication, heart defect complexity, cyanosis, and trisomy 21 seem to also play a fundamental role, as seen in our series.

Medication such as the several drugs used frequently in the setting of CHD have known nephrotoxicity. One such class of drugs are angiotensin-converting enzyme inhibitors, which are used to control blood pressure, improve ventricular function, and prevent pathologic ventricular remodeling. Despite the beneficial use of this treatment, caution should be exercised with the goal of minimizing acute kidney injury. Also, the inappropriate use of loop diuretics may exacerbate renal hypoperfusion through vasodilatation and excessive diuresis, resulting in renal failure. Likewise, non-steroidal anti-inflammatories frequently used for analgesia in patients that undergo congenital heart repairs may have a negative impact on renal function as the kidneys in patients with heart failure are more dependent on prostaglandins in the maintenance of renal blood flow [17,25].

CHD complexity, as it frequently associates with multiple surgical interventions, the use of nephrotoxic contrasts agents during percutaneous procedures, ventricular dysfunction, or the use of diuretic treatment [26]. In fact, a low cardiac output secondary to systemic ventricular dysfunction may result in intraglomerular hemodynamic changes, renal hypoperfusion, sympathetic nervous and renin-angiotensin-aldosterone system activation, and renal dysfunction.

Cyanosis, as a long-lasting hypoxemia, contributes to the appearance of glomerulopathy and proteinuria [27]. In this context, in a study conducted to evaluate the prevalence of renal dysfunction in adults with CHD, Dimopoulos et al. [19] found that patients with cyanosis exhibited more significantly reduced kidney function compared to those without cyanosis. Among these, cyanotic patients with Eisenmenger syndrome had the lowest glomerular filtration rate. Similarly, in our series, cyanosis was related to a worse renal function. Different factors can explain these facts. On the one hand, chronic hypoxia entails low oxygen levels, which may damage renal microcirculation, reducing their ability to filter blood efficiently, and on the other hand, hyper-viscosity, secondary to erythrocytosis, which may increase shear stress and the intraglomerular release of nitric oxide, leading to ischemia and further damage to the kidney tissue.

Down syndrome patients are also at risk of kidney impairment. This is likely due to an increased risk of congenital kidney and urological malformations, more frequently associated comorbidities at risk of kidney dysfunction (such as prematurity, intrauterine growth retardation, and low birth weight), and more frequent lower urinary tract dysfunction [28]. Additionally, autoimmune conditions and a higher prevalence of atherosclerotic risk factors further contribute to renal dysfunction. Added to this is the fact that almost half of patients with Down syndrome have CHD and that older individuals with Down syndrome did not benefit from CHD repair as many years ago, they were less likely to receive surgical treatment, resulting, therefore, in the development of Eisenmenger syndrome and secondary cyanosis, which carries an additional risk of kidney dysfunction [29].

In relation to whether low plasma iron levels are a risk factor for developing kidney failure or on the contrary, are a side effect of kidney deterioration, we can say that several risk factors contribute to absolute and functional iron deficiency in CKD, including blood losses, impaired iron absorption, and chronic inflammation. Also, the need for an increased circulating hemoglobin mass in CHD patients with associated cyanosis puts a severe stress on their endogenous and dietary iron supplies, resulting in relative iron deficiency anemia [30,31]. Therefore, iron deficiency anemia and cyanosis may go hand in hand when explaining the increased risk of kidney disease in patients with CHD.

### 4.3. Cardiovascular Mortality and Renal Failure in Patients with CHD

Many studies have shown consistently an increased risk for cardiovascular disease and all-cause mortality in the general population [32,33]. However, relatively few studies have investigated the association between CKD and mortality in patients with CHD. In this context, Parikh et al. [34] found that the risk of mortality and end stage kidney disease was high in children who underwent surgical repair for CHD compared to the general population. Similarly, Dimopoulous et al. estimated that 9% of adult patients with CHD had moderate or severely impaired renal function and as a result, they had an additional adjusted three-fold increase in mortality risk. In our series, we also found that patients with CHD and renal failure had a worse outcome with an almost seven-fold increase in the prevalence of cardiovascular mortality when compared with the adult CHD patients with a GFR above 60 mL/min. However, factors such as the patient’s age were more important, losing renal dysfunction, and its significance in multivariate survival analysis.

### 4.4. Limitations of the Study

Although socioeconomic inequalities, especially during the years of crisis, may have an external influence on the prevalence of CKD due to the inequity in access to health, we believe that this fact has not had a relevant influence on our series; both groups (CHD patients and the control population) were obtained from the same geographical area and the health system in Spain is based on the principles of universality, free of charge, equity, and justice in financing, which makes it accessible to the entire general population [35]. Also, we did not determine factors contributing to renal failure such as the number of cardiac surgeries and the impact of surgical procedures, the number of percutaneous procedures with the consequent use of potentially nephrotoxic contrast, or the prematurity or low birth weight which may further increase the likelihood of renal complications. Likewise, our study had a single-center design; we excluded younger patients, and we did not perform renal imaging tests with the consequent difficulty of determining whether other factors contribute to the deterioration of renal function. Also, the limited number of cardiovascular deaths observed during the follow-up period, which is characteristic of younger populations, constrained our ability to capture a larger number of events. Nevertheless, we believe that our sample size is sufficient to establish a correlation between GFR concentrations and cardiovascular outcomes in patients with CHD. It is also important to note that patients with CHD are a heterogeneous group, which may complicate the ability to draw definitive conclusions regarding their outcomes. Despite these limitations, we contend that the inclusion of a substantial number of patients with CHD in our study, along with the opportunity to compare them to a control population, enhances our understanding of the potential role of renal function as a predictor of cardiovascular events in this patient population. In any case, further studies with a larger number of patients, a longer-term follow-up, and a greater number of cardiovascular events will be necessary to determine risk factors for CKD among CHD patients and how renal insufficiency influences their prognosis.

## 5. Conclusions

In conclusion, in our series, we found a much higher prevalence of CKD among patients with CHD than in the control population, with the older, cyanotic, and Down syndrome CHD patients being at a higher risk. Although CKD was a predictor of survival in the Kaplan and univariate Cox regression analysis, after including well known clinical factors such as age, CHD complexity, or NT-pro-BNP levels, this significance was lost. Understanding the factors that impact renal function in patients with CHD is essential for the effective management and treatment of these patients.

## Figures and Tables

**Table 1:** Types of CHD according to their anatomical complexity.

	CHD Complexity			
Types of CHD	Mild	Moderate	Great	Total
Ventricular septal defect	105	0	6	111
Atrial septal defect	76	0	8	84
Pulmonary valve disease	68	0	1	69
Aortic coarctation	0	55	0	55
Repaired tetralogy of Fallot	0	55	0	55
Bicuspid aorta	31	0	0	31
Dextro-Transposition of the great arteries (d-TGA)	0	0	30	30
Partial atrioventricular septal defect (p-AVSD)	0	20	3	23
Subaortic membrane	0	20	0	20
Complete atrioventricular septal defect (c-AVSD)	0	18	4	22
Aortic stenosis	15	0	0	15
Ductus arteriosus	14	0	0	14
Mitral valve prolapse	14	0	0	14
Double outlet right ventricle (DORV)	0	0	16	16
Single ventricle	0	0	12	12
Congenitally corrected transposition of the great arteries (cc-TGA)	0	0	15	15
Aortic regurgitation	10	0	0	10
Pulmonary atresia	0	0	11	11
Subvalvular and supravalvular pulmonary stenosis	0	8	0	8
Patent foramen ovale	7	0	0	7
Tricuspid atresia	0	0	6	6
Ebstein anomaly of the tricuspid	0	6	0	6
Mitral regurgitation	5	0	0	5
Subvalvular and supravalvular aortic stenosis	0	4	0	4
Anomalous pulmonary venous return	0	3	2	5
Truncus arteriosus	0	0	2	2
Marfan syndrome	2	0	0	2
Systemic venous anomaly	1	0	0	1
Vascular ring	1	0	0	1
Idiopathic pulmonary artery aneurysm	1	0	0	1
Aortopulmonary fistula	1	0	0	1
Systemic arteriovenous fistula	0	0	1	1
Total	351	189	117	657

CHD: congenital heart disease.

**Table 2:** Demographic, clinical, and blood test data of patients with CHD and the control group.

	Control	CHD	*p* *
CHD patients, n	1954	657	
Age, years	33 (16–51)	30 (17–62)	0.390
Sex (male), n	1051 (54)	373 (57)	0.171
Arterial hypertension, n	186 (9)	84 (13)	0.017
Diabetes mellitus, n	48 (3)	33 (5)	0.001
Dyslipidemia, n	481 (25)	187 (28)	<0.001
Smoking, n	33 (2)	15 (2)	<0.001
Blood test			
Glucose, mg/dL	94 (81–155)	93 (78–115)	0.010
Creatinine, mg/dL	0.7 (0.5–1.0)	0.9 (0.5–1.2)	<0.001
GFR, mL/min/1.73 m^2^	112 (83–154)	94 (60–169)	<0.001
GFR (<60 mL/min/1.73 m^2^), n	3 (0.2)	30 (4.6)	<0.001
Hemoglobin, mg/dL	14 (12–17)	15 (12–17)	0.002
Total bilirubin, mg/dL	0.6 (0.3–1.4)	0.7 (0.3–2.1)	<0.001
Total cholesterol, mg/dL	177 (122–249)	161 (108–232)	<0.001
LDL cholesterol, mg/dL	104 (60–163)	92 (48–157)	<0.001
HDL cholesterol, mg/dL	51 (36–75)	49 (32–70)	<0.001
ALT, IU/L	18 (9–58)	18 (10–50)	0.843
AST, IU/L	21 (14–43)	22 (14–43)	0.002
Hs-CRP, mg/dL	0.2 (0.0–1.4)	0.2 (0.0–1.5)	0.692
Medical treatment			
Antiplatelet, n	25 (1)	68 (10)	<0.001
Oral anticoagulation, n	5 (0.2)	93 (14)	<0.001
Betablockers, n	47 (2)	97 (15)	<0.001
ACE inhibitors, n	63 (3)	60 (9)	<0.001
ARBs, n	71 (4)	35 (5)	0.106
Calcium channel blockers, n	28 (1)	23 (3)	0.001
Loop diuretics, n	61 (3)	92 (14)	<0.001
Oral iron, n	80 (4)	32 (5)	<0.001
Statins, n	138 (7)	50 (8)	0.523

CHD: congenital heart disease, n: number of patients, GFR: glomerular filtration rate, ALT: alanine aminotransferase, AST: aspartate aminotransferase, Hs-CRP: high sensitivity C-reactive protein, ACE: angiotensin converting enzyme, ARBs: angiotensin receptor blockers. The data are expressed as median and (5–95) percentiles and as a number and percentage. * Categorical variables are evaluated by the Pearson chi-square test, continuous data with normal distribution are compared with Student’s *t*-test, and continuous data without normal distribution with the Mann–Whitney test.

**Table 3:** Demographic, clinical, and blood test data of CHD patients according to CKD.

	CHD Patients		*p* *
	Without CKD	With CKD	
CHD patients, n	627	30	
Age, years	30 (18–60)	53 (27–89)	<0.001
Sex (male), n	363 (57.9)	10 (33.3)	0.008
BMI, kg/m^2^	23 (18–35)	24 (20–33)	0.029
Great CHD complexity, n			<0.001
Mild	344 (55)	7 (23)	
Moderate	182 (29)	7 (23)	
Great	101 (16)	16 (54)	
NYHA functional class (≥2), n	22 (3)	9 (37)	<0.001
Arterial hypertension, n	75 (12)	9 (30)	0.004
Diabetes mellitus, n	26 (4)	7 (23)	<0.001
Dyslipidemia, n	95 (15)	12 (40)	<0.001
Smoker, n	31 (5)	0 (0)	0.008
Blood test			
Glucose, mg/dL	93 (79–115)	98 (71–147)	0.151
Creatinine, mg/dL	0.9 (0.5–1.1)	1.3 (1.0–2.2)	<0.001
GFR, mL/min/1.73 m^2^	95 (70–170)	50 (30–59)	<0.001
Hemoglobin, mg/dL	15 (12–17)	14 (9–122)	0.994
Total bilirubin, mg/dL	0.7 (0.3–2.0)	0.9 (0.3–3.2)	0.269
Total cholesterol, mg/dL	160 (109–232)	167 (94–258)	0.755
LDL cholesterol, mg/dL	92 (48–155)	103 (31–170)	0.496
HDL cholesterol, mg/dL	49 (32–71)	47 (24–165)	0.127
ALT, IU/L	18 (10–51)	19 (9–317)	0.950
AST, IU/L	32 (14–43)	25 (13–229)	0.1000
NT-pro-BNP, pg/mL	63 (0–999)	757 (39–2148)	<0.001
Iron, µg/dL	81 (25–153)	44 (15–93)	0.001
Ferritin, ng/mL	36 (6–191)	25 (4–56)	0.176
Hs-CRP, md/dL	0.15 (0.0–1.5)	0.5 (0.1–1.6)	<0.001
Treatment			
Antiplatelet, n	63 (10)	5 (17)	0.244
Oral anticoagulation, n	83 (13)	13 (43)	<0.001
Beta-blockers, n	89 (14)	8 (27)	0.042
ACE inhibitors, n	55 (9)	5 (17)	0.121
ARBs, n	30 (5)	5 (17)	0.003
Calcium channel blockers, n	21 (3)	2 (7)	0.308
Loop diuretics, n	75 (12)	17 (57)	<0.001
Statins, n	43 (7)	7 (23)	0.001
Oral iron, n	28 (4)	4 (13)	0.022
Mechanical valve prosthesis, n	28 (4)	1 (3)	0.440
Systemic ventricular dysfunction ^#^, n	103 (16)	5 (17)	0.976
Cyanosis, n	40 (6)	13 (43)	<0.001
Arterial pulmonary hypertension, n	31 (5)	12 (40)	<0.001
Down syndrome, n	35 (6)	7 (23)	<0.001
Cardiovascular mortality, n	21 (3)	10 (33)	<0.001

CHD: congenital heart disease, CKD: chronic kidney disease, BMI: body mass index, NYHA: New York Heart Association, GFR: glomerular filtration rate, ALT: alanine aminotransferase, AST: aspartate aminotransferase, NT-pro-BNP: NT-pro-brain natriuretic peptide, Hs-CRP: high sensitivity C reactive protein, ACE: angiotensin converting enzyme, ARBs: angiotensin receptor blockers, ^#^ moderate to severe systemic ventricular dysfunction. The data are expressed as median and (5–95) percentiles and as a number and percentage. * Categorical variables are evaluated by the Pearson chi-square test, continuous data with normal distribution are compared with Student’s *t*-test, and continuous data without normal distribution with the Mann–Whitney test.

**Table 4:** Binary logistic regression analyses in CHD patients to predict CKD.

	OR (Crude) (95% CI)	*p*	OR (Adjusted) (95%CI)	*p*
Age, years	1.07 (1.05–1.10)	<0.001	1.54 (1.04–1.28)	0.009
BMI, Kg/m^2^	0.12 (1.05–0.98)	0.119		
NYHA (≥2)	13.4 (5.3–33.9)	<0.001	6.56 (0.21–128)	0.279
Diabetes mellitus, yes	7.0 (2.8–17.9)	<0.001	0.26 (0.01–4.75)	0.364
Dyslipidemia, yes	3.7 (1.7–8.0)	0.001	19.8 (1.35–301.1)	0.031
Sex, male	2.7 (1.27–5.99)	0.010	6.75 (0.45–100.1)	0.165
Arterial hypertension	3.1 (1.4–7.2)	0.006	5.82 (0.40–83.6)	0.195
NT-pro-BNP, pg/mL	1.001 (1.00–1.001)	0.002	1.00 (0.99–1.00)	0.728
Iron, µg/dL	0.97 (0.96–0.99)	0.002	0.96 (0.96–0.93)	0.048
Hs-CRP,	1.09 (0.89–1.34)	0.405		
Cyanosis, yes	11.2 (5.1–24.7)	<0.001	25.7 (1.60–411.8)	0.022
PAH, yes	13.6 (5.9–31.6)	<0.001	1.46 (0.167–12.89)	0.729
Down síndrome, yes	5.0 (2.0–12.6)	0.001	46.8 (8.09–2710)	0.003

CHD: congenital heart disease, CKD: chronic kidney disease, BMI: body mass index, NYHA: New York Heart Association, NT-pro-BNP: NT-pro-brain natriuretic peptide, Hs-CRP: high sensitivity C reactive protein, PAH: pulmonary arterial hypertension, OR: odds ratio, CI: confidence interval.

**Table 5:** Univariate and multivariate Cox regression analysis of variables associated with cardiovascular mortality in patients with CHD.

	Univariate Analysis		Multivariate Analysis	
	HR (95% CI)	*p*	HR (95% CI)	*p*
Age, years	1.06 (1.04–1.09)	<0.001	1.05 (1.02–1.08)	0.002
CHD complexity ^a^	3.30 (1.53–7.14)	0.002	2.19 (0.71–6.78)	0.172
NYHA class ^b^	3.62 (1.38–9.50)	0.009	1.27 (0.41–3.92)	0.672
Diabetes mellitus, yes	2.04 (0.61–6.86)	0.250		
NT-pro-BNP ^c^	7.42 (1.74–31.6)	0.007	4.71 (0.60–37.08)	0.141
CKD	9.71 (4.32–21.08)	<0.001	1.90 (0.57–6.36)	0.295

CHD: congenital heart disease, NYHA: New York Heart Association functional class, NT-pro-BNP: NT-pro-brain natriuretic peptide, CKD: chronic kidney disease, HR: hazard ratio, CI: confidence interval. CHD complexity, NYHA class, NT pro-BNP, and CKD are treated as binary variables: ^a^ CHD mild and moderate vs. great complexity, ^b^ NYHA class I vs. patients with class ≥ 2, ^c^ NT-pro-BNP if levels were below 125 or ≥125 pg/mL, and CKD if GFR (glomerular filtration rate) concentrations was ≥ or <60 mL/min/1.73 m^2^.

## Data Availability

The participants of this study did not give written consent for their data to be shared publicly, so due to the sensitive nature of the research, supporting data are not available.
